# Predicting and explaining life satisfaction among older adults using tree-based ensemble models and SHAP: Evidence from the digital divide survey

**DOI:** 10.1371/journal.pone.0337938

**Published:** 2025-12-16

**Authors:** Haiyan Kong, Hualong Fang, Guihua Zhang

**Affiliations:** 1 School of Business, Xinyang Normal University, Xinyang, China; 2 Dabie Mountain Economic and Social Development Research Center, Xinyang, China; 3 Department of Global Business, Chungbuk National University, Cheongju, Republic of Korea; National Cheng Kung University, TAIWAN

## Abstract

As digital transformation continues to penetrate various sectors of society, the issue of the digital divide has become increasingly prominent. Against the backdrop of accelerating population aging, the barriers that older adults face in accessing and utilizing digital information have exerted a profound impact on their quality of life. This study employs tree-based ensemble learning algorithms to predict and identify the key factors of the digital divide that influence life satisfaction among older adults. It also evaluates the predictive performance of these models, thereby providing interpretive insights into the impact of the digital divide on subjective well-being. Using original data from the ‘2023 Report on Digital Information Divide Survey’ conducted by the National Information Society Agency of South Korea, this study constructs an analytical framework that integrates both predictive capability and interpretability. First, the XGBoost model is employed to conduct feature importance analysis, identifying 15 key variables that are highly influential in predicting life satisfaction. These variables are further examined using the SHAP method to provide interpretive insights into their contributions. Subsequently, multiple tree-based ensemble learning algorithms—including Random Forest, XGBoost, LightGBM, and CatBoost—are applied to compare their predictive performance. The results indicate that variables related to technological self-efficacy, digital information literacy, social capital, experience and perception of AI services, and household monthly income are significant predictors of life satisfaction among older adults. Among the models tested, CatBoost demonstrates superior overall predictive accuracy, suggesting its effectiveness in forecasting life satisfaction in this demographic. This study expands the application of machine learning in areas such as aging research and the digital divide and proves the effectiveness of ensemble learning algorithms in predicting digital divide factors that affect the life satisfaction of older adults. This approach provides a novel and powerful methodological for addressing complex social problems. Moreover, the study uncovers the structural configuration of key digital information factors associated with life satisfaction, offering data-driven insights into the mechanisms through which the digital divide influences well-being. These results have practical implications for enhancing digital inclusion, improving adaptability among older adults, and fostering a stronger sense of participation and happiness in digital society.

## Introduction

With the widespread application of Information and Communication Technology (ICT) in everyday life, digital technologies have deeply penetrated various sectors such as financial transactions, e-commerce, and self-service terminals (e.g., kiosks), profoundly transforming both societal operations and individual lifestyles. However, alongside the rapid diffusion of these technologies, the digital information divide has been exacerbated, emerging as a significant risk factor contributing to socio-economic inequalities. Existing research has highlighted that the digital divide not only impedes access to information and digital services but also constrains individuals’ opportunities for social participation and overall well-being, thereby reinforcing structural disparities across generations, regions, and socio-economic groups [1,2].

Global demographic changes further complicate this challenge. As of 2023, the global population aged 60 and above has exceeded 1.3 billion, accounting for 17% of the total population; according to United Nations projections, this proportion will rise to over 22% by 2050, with the number of older persons doubling to 2.1 billion [3].In South Korea, this trend is particularly pronounced: by 2025, individuals aged 65 and over are expected to comprise 20.3% of the national population, and this share is projected to surpass 30% by 2036, marking the transition into a ‘super-aged society’ [4]. These demographic shifts highlight the urgent need to address older adults’ adaptability to digital environments and their degree of social inclusion, which directly influence the formulation of welfare policies and the inclusive design of public services, thereby emerging as a central issue in aging governance in the digital era.

However, due to the physical and cognitive decline associated with aging, older adults often face significant barriers in adapting to new technologies and accessing information. As a result, they remain in a state of digital marginalization and are widely recognized as a digitally disadvantaged group. Recent empirical evidence reinforces this perspective. Jiao et al. [5] found a positive correlation between lower frequency of Internet use and cognitive decline among older adults in China. Similarly, Yang et al. [6] reported that U.S. older adults with limited access to digital media exhibited poorer self-reported health and weaker information acquisition capacity. Fang et al. [7] further highlighted the critical role of self-efficacy and technology-related beliefs in shaping online health information-seeking behavior.

The rapid digitalization of public services has further intensified these challenges, as more and more service processes are being transferred to digital platforms. Older adults, constrained by limited cognitive abilities, digital literacy, and technological competence, often face considerable difficulties in accessing government services and essential information. In some cases, they risk being excluded from even the most basic public service systems, thereby undermining their civic rights and social participation [8,9]. The COVID-19 pandemic magnified these inequalities: with lockdowns and the suspension of offline services, older adults were compelled to rely on digital channels for healthcare, communication, and daily necessities. In this context, disparities in digital adaptability were brought into sharp relief, underscoring the decisive role of technology in shaping older adults’ quality of life and degree of social integration [10-, 11,12]. While the pandemic accelerated the digitalization of public services, it also exposed the vulnerability of older populations in terms of their ‘digital emergency capacity’. As a result, mitigating digital inequalities and preventing social exclusion due to technological barriers have become pressing priorities in the governance of aging societies.

Recent research on active aging suggests that the appropriate integration of digital technologies can significantly enhance older adults’ social participation, self-actualization, and emotional connectedness, thereby improving their overall quality of life [13,14]. In particular, digital tools such as smartphones and the internet have provided new avenues for older adults to access services, build social networks, and engage in lifelong learning [15]. For instance, through online communities, telemedicine platforms, and digital education resources, older adults can strengthen their independence and psychological resilience [16]. Moreover, using social media and email to maintain social connections has been shown to enhance their social capital and life satisfaction [17]. However, most existing studies rely on traditional regression models or mediation analyses, which generally assume linear or low-order relationships among variables. These studies also tend to focus on a limited number of influencing factors, failing to capture the complex, multidimensional structures that underlie subjective well-being in real-life settings [13].

In recent years, machine learning methods, as data-driven analytical approaches, have demonstrated significant advantages over traditional techniques in predicting social behaviors and identifying key variables. Prior studies have shown that machine learning can be applied to predict digital divide risks among older adults [18], uncover nonlinear relationships between age and life satisfaction [19], and, in some cases, outperform traditional regression models in predicting subjective well-being among older adults [20]. While conventional statistical approaches emphasize model structure and causal inference—making them suitable for theory-driven research—machine learning prioritizes pattern recognition and predictive accuracy, especially in high-dimensional, nonlinear, and complex interaction settings [21,22]. Among machine learning techniques, tree-based ensemble learning methods have shown particular promise in analyzing structured social survey data. These methods are well-regarded for their ability to handle overfitting, capture high-order interactions, and have been widely applied in fields such as social sciences, health research, and public policy [20,23]. Nonetheless, a key limitation of such models lies in their interpretability; the ‘black-box’ nature of model outputs often makes it difficult to intuitively understand the pathways through which variables affect outcomes. To address this limitation, interpretable machine learning techniques such as SHAP (SHapley Additive exPlanations) have been developed. SHAP originates from the Shapley value theory in cooperative game theory and can quantify the marginal contribution of each input variable to model prediction, thereby enhancing the interpretability of complex models [24].By integrating ensemble learning with SHAP, researchers can simultaneously achieve strong predictive performance and model interpretability, offering empirical evidence to support both theoretical development and policy interventions.

Drawing on large-scale data from the ‘2023 Report on Digital Information Divide Survey’ in South Korea, this study employs an interpretable machine learning framework that combines tree-based ensemble models with the SHAP method to identify key digital divide factors influencing life satisfaction among older adults. The primary objective of this research is to develop a robust and interpretable predictive model, providing both theoretical insights and practical recommendations to inform digital inclusion policies in aging societies.

## Literature Review

### Digital divide and digital literacy among older adults

With the widespread diffusion of Information and Communication Technology (ICT), the digital divide has increasingly become a critical dimension in assessing social equity and digital inclusion. The digital divide not only refers to inequalities in infrastructure and technological access but has also evolved into a complex structural issue rooted in disparities of cognitive capacity, skill levels, and practical application [25]. Within this discourse, Digital information literacy is regarded not only as a determinant of the digital divide but also as an integral component of the divide itself [26].

Digital information literacy is a multidimensional concept encompassing a range of cognitive and operational competencies, including the ability to access, comprehend, evaluate, and apply information. This concept integrates elements of information literacy, digital literacy, and ICT-related skills [27]. In a society increasingly structured around technological systems, an individual’s level of digital literacy largely determines their opportunities and performance in education, employment, health, and social participation.

In the digital era, digital information literacy is increasingly recognized as both a fundamental driver of national competitiveness and a critical factor influencing individual well-being and quality of life [28]. This trend is particularly salient in the context of aging societies. Constrained by physical decline, rising cognitive burdens, and limited educational backgrounds, older adults are often positioned at the margins of digital society, becoming a prototypical digitally disadvantaged group [1]. Van Dijk [29] similarly argued that the digital divide is not a matter of access alone but constitutes a multifaceted process involving motivational access, physical access, skills access, and usage access [30]. This framework highlights the multiple roots of digital inequality, aligning closely with the real challenges faced by older adults in terms of technology anxiety, lack of autonomy, and barriers to the use of smart devices and digital services. Building on this perspective, the present study incorporates digital information literacy as a core explanatory variable within the mechanism of the digital divide, aiming to examine its pathways of influence on older adults’ life satisfaction.

A growing body of empirical research has demonstrated that digital information literacy is closely associated with subjective well-being among older adults. Those with higher levels of digital literacy are generally more capable of establishing new social networks through the internet, participating in online learning and remote communication, thereby alleviating loneliness, reducing depressive symptoms, and improving life satisfaction [31-, 32,33]. Although some studies have noted that older adults may experience psychological resistance to digital tools due to the perceived complexity of IT systems, studies have shown that providing appropriate guidance and support for the use of digital devices, internet platforms, and related technologies can effectively enhance older adults’ sense of self-efficacy, promote social engagement, and improve their quality of life [31,33].

According to the ‘2023 Report on Digital Information Divide Survey’ by the National Information Society Agency (NIA) of South Korea, older adults continue to lag significantly behind younger and middle-aged groups in terms of digital access, technological capabilities, and practical utilization skills [34]. This gap not only reflects the lack of digital literacy education tailored to older adults within formal education systems, but also exposes their systemic disadvantages in key areas such as online information retrieval, electronic payments, digital administrative services, and virtual social interactions [18]. In the context of accelerating global population aging, failure to address this issue may further widen the digital divide and intensify the risks of social marginalization among older adults in the future. Therefore, identifying key digital divide factors that affect the quality of life of older adults has become an essential research priority in addressing the challenges of fostering an inclusive and active aging society.

### Life satisfaction

Life satisfaction is commonly defined as the degree of subjective contentment individuals perceive in various aspects of their lives, serving as one of the core indicators for assessing subjective well-being and quality of life [35]. This concept not only reflects individuals’ overall evaluations of their living conditions but also encompasses their perceptions of self-actualization, daily positive experiences, and psychological comfort. For older adults, life satisfaction is shaped by a wide range of factors, including physical health status, social support networks, financial security, residential environment, and opportunities for social participation [36,37].

In recent years, with the increasing penetration of ICT, digital capabilities have been progressively incorporated into the emerging framework of factors influencing life satisfaction. Empirical studies have shown that older adults who are adept at using digital devices to access and utilize information tend to exhibit stronger senses of self-efficacy, social connectedness, and control over their lives [38,39]. In particular, amid the widespread diffusion of the internet, older adults are increasingly engaging in social activities, maintaining interpersonal relationships, and accessing healthcare and government services through digital means. Such behaviors have been strongly linked to higher levels of subjective well-being and life satisfaction [40,41].

Against the backdrop of simultaneous population aging and societal digitalization, older adults not only face physiological and psychological changes associated with aging but are also required to adapt to technology-driven societal norms and systems. Therefore, identifying and understanding the digital information usage factors that influence life satisfaction among older adults carries significant theoretical and practical implications. Theoretically, such inquiry contributes to the advancement of active aging research. Practically, it provides critical insights for promoting a digitally inclusive society. In particular, enhancing digital engagement among older adults is increasingly viewed as a crucial pathway for improving their life satisfaction and fostering greater social integration, especially as public services, healthcare systems, and social governance become progressively digitalized [15,42].

### Applications of machine learning

Machine learning (ML), as one of the core branches of artificial intelligence, focuses on automatically learning patterns from data and making predictions through algorithms [43]. Unlike traditional statistical methods, which emphasize parameter estimation and causal inference, machine learning prioritizes predictive performance and adaptability to data. It has demonstrated considerable advantages in modeling high-dimensional, nonlinear, and complex interaction relationships [21,22]. Significant progress has been made in applying machine learning across various practical domains, including disease diagnosis [44], financial fraud detection [45], natural language processing [46], and image recognition [47]. Nevertheless, conventional machine learning algorithms still face challenges related to data imbalance, variable heterogeneity, and model generalizability [48]. To overcome these limitations, ensemble learning methods have been developed to enhance the stability and accuracy of the model by combining the prediction outputs of multiple weak learners. Ensemble learning has shown its excellent ability in analyzing complex multidimensional social data, providing a powerful solution to the inherent complexity and variability of such datasets [23].

Although machine learning techniques have been widely applied in fields such as medicine, business, and public opinion analysis, their use in uncovering the complex predictive mechanisms underlying life satisfaction among older adults remains limited. Existing studies have predominantly focused on employing traditional algorithms to conduct classification and regression analyses of older adults’ internet usage behaviors or successful aging outcomes [43,49,50], or have been confined to examining linear relationships between variables [19,51]. Given that the determinants of life satisfaction are often multidimensional, interactive, and nonlinear in nature, relying solely on conventional models is insufficient to fully capture the complexity of real-world phenomena.

To address this gap, the present study introduces several tree-based ensemble learning methods. Tree-based models are among the most widely used non-linear models, and in the field of machine learning, ensemble methods that integrate multiple tree models are capable of constructing more robust predictive frameworks. Prior research has demonstrated that, compared to linear models and even certain deep learning approaches, tree-based methods often achieve superior performance in prediction tasks involving structured data [52,53–55]. To comprehensively evaluate predictive performance, this study employs four mainstream tree-based ensemble models, including Random Forest, XGBoost, LightGBM, and CatBoost. These algorithms have demonstrated superior performance in handling structured data and are particularly well-suited for high-dimensional prediction tasks and modeling complex interrelationships among features.

### Random forest

The Random Forest algorithm, proposed by Breiman, utilizes decision trees as its base learners. It employs bootstrap sampling to generate multiple sub-datasets from the original training data, and a separate decision tree model is trained on each sub-dataset. The final prediction is obtained by aggregating the outputs of all trees through a majority voting mechanism for classification tasks or averaging for regression tasks [56]. This algorithm is particularly effective in handling high-dimensional data and mitigating overfitting, thereby ensuring robust predictive performance. Due to these advantages, Random Forest has been widely applied across various fields, including classification and regression tasks [57].

### Gradient boosting

The Gradient Boosting algorithm is commonly used for classification and regression tasks. This method assigns greater weights to instances with larger prediction errors from previous learners, enabling the model to focus more effectively on correcting prior mistakes and improving classification performance [58]. In general, Gradient Boosting tends to outperform bagging-based algorithms, such as Random Forest, in terms of predictive accuracy. However, it requires longer training times and is more susceptible to overfitting [59]. In this study, widely used Gradient Boosting variants, including XGBoost, LightGBM, and CatBoost, are adopted.

- **XGBoost**

XGBoost developed by Chen and Guestrin, was designed to enhance stability and improve training speed on large-scale datasets [60]. The algorithm supports parallel and distributed computing, offering excellent scalability. Moreover, it can effectively handle missing values and performs well on sparse datasets [53]. XGBoost also incorporates a regularization mechanism that penalizes overly complex models, thereby constraining tree depth and preventing overfitting. Due to these advantages, XGBoost has been widely applied across various fields.

- **LightGBM**

LightGBM, developed by Microsoft in 2017, demonstrates superior performance compared to XGBoost in terms of speed, computational efficiency, and memory usage [61]. To effectively reduce data processing time, LightGBM does not scan all data points when searching for split points at each tree node. Instead, it employs Gradient-based One-Side Sampling (GOSS), which focuses on data points with large gradients. In addition, it utilizes the Exclusive Feature Bundling (EFB) technique to merge mutually exclusive features into a single feature, thereby reducing the number of input variables, accelerating the training process, and minimizing memory consumption [61]. These optimizations make LightGBM particularly well-suited for large-scale datasets and significantly improve computational efficiency.

- **CatBoost**

CatBoost is specifically designed to handle categorical data and address the problem of overfitting. Traditional Gradient Boosting algorithms typically use the entire training dataset to iteratively compute residuals, which often leads to overfitting on training data [55]. CatBoost mitigates this risk by generating ordered subsets of the training data and calculating residuals within these subsets, effectively reducing overfitting [62]. Due to its strong capability for processing categorical features, CatBoost has been widely applied in various domains, including natural language processing (NLP) and financial forecasting.

Furthermore, to enhance model interpretability, this study incorporates SHAP, an interpretability technique that has gained increasing attention in recent years. SHAP quantifies the marginal contribution of each input variable to the model’s prediction, thereby offering interpretive insights into otherwise “black-box” models [24]. SHAP is recognized for its consistency and local accuracy, and it can be applied to explain predictions from any complex model. It has been widely adopted in fields such as finance, healthcare, energy, and public administration for variable importance analysis46 [63,64,65]. In this study, SHAP is employed to elucidate the direction and magnitude of each feature’s contribution to the model’s output. This approach not only identifies the key factors influencing life satisfaction but also provides a transparent depiction of the interaction mechanisms and directional effects among variables. The findings offer both theoretical foundations and empirical evidence to inform the development of targeted digital inclusion strategies for aging societies.

## Materials and methods

### Data selection and preprocessing

This study utilized cross-sectional data from the Digital Divide Survey conducted by the National Information Society Agency (NIA) of South Korea between September and December 2023. Since 2002, this survey has been administered annually as a nationwide statistical project by the Korean government, with the aim of systematically assessing the digital divide between the general population and information-vulnerable groups, including low-income individuals, persons with disabilities, farmers and fishers, and older adults. A multi-stage stratified sampling design was employed, with square-root proportional allocation to ensure representativeness across key sociodemographic variables such as region, gender, age, educational attainment, and income.

The analytical focus of this study was on older adults. From the original sample of 2,300 respondents, which covered youth, middle-aged, and older cohorts, individuals aged 65 years and above were classified as older adults, in accordance with the eligibility criteria for welfare services (e.g., health check-ups, senior benefits) defined in the National Basic Living Security Act. To align with the study’s objectives, we further restricted the sample to those aged 65 and older who had prior Internet experience and provided valid responses, yielding a final analytic sample of 869 older adults. The demographic characteristics of the study population are presented in Table 1.

**Table 1:** Characteristics of demographic.

Classification		N	%
Gender	Male	419	48.22
	Female	450	51.78
Age	65-74	663	76.29
	More 75	206	23.71
Education	below primary school	182	20.94
	middle school grad	289	33.26
	high school grad	345	39.70
	More college	53	6.10
Monthly house Income (KRW)	below 2,000,000	269	34.06
	2,000,000-3,990,000	385	44.3
	4,000,000-5,990,000	137	15.77
	More 6,000,000	51	5.87
Physical Condition	Healthy	855	98.39
	Disabled	14	1.61
Region of Residence	Urban	750	86.31
	Rural	119	13.69
living alone	living alone	158	18.18
	living together	711	81.82

During the data preprocessing stage, variables containing personally identifiable information (e.g., ID numbers, addresses) were first removed. Subsequently, missing-value analysis was conducted, which revealed a relatively high proportion of missingness in certain variables among the older adult subgroup. For case-level missingness within the retained variables, we adopted a listwise deletion strategy. Such missingness primarily stemmed from structural missingness (e.g., questions related to employment or child-rearing that were not applicable to retired or childless respondents) and logical missingness (e.g., responses such as “don’t know” or skipped answers due to memory limitations or knowledge gaps), rather than random missingness. Although these variables may be meaningful in the overall population, they lacked applicability and measurement validity within the elderly sample that constitutes the focus of this study. Considering variable relevance, missingness rates, and modeling feasibility, variables with more than 30% missingness and limited representativeness or explanatory power for older adults were excluded to avoid estimation bias caused by structural missingness. Thereafter, categorical variables were encoded, and continuous variables were standardized prior to analysis.

**Fig 1:**
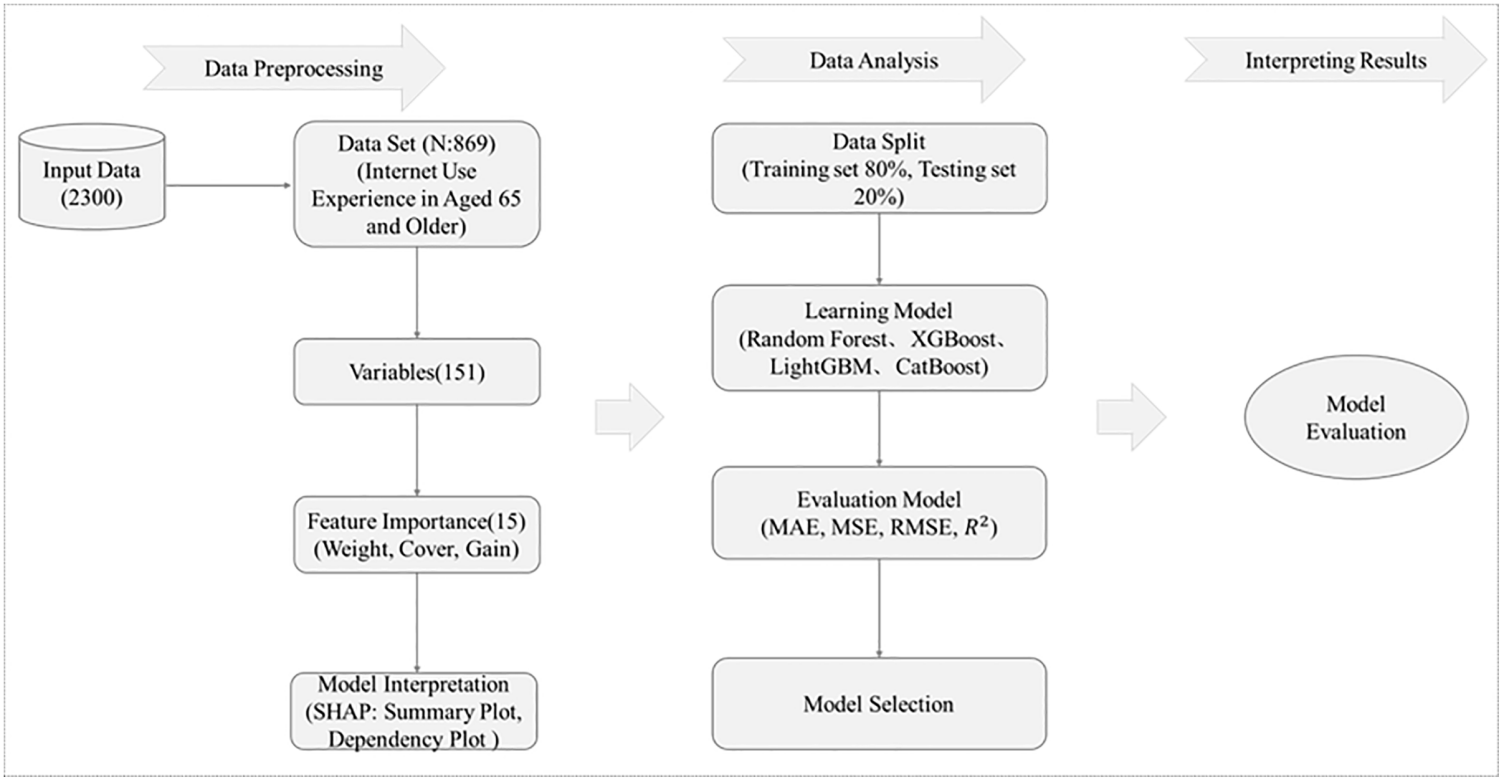
Research process.

Subsequently, the XGBoost model is applied to conduct feature importance analysis, identifying key digital divide factors that are significantly associated with life satisfaction. To enhance model interpretability, the SHAP method is incorporated to quantify the marginal contribution and directional influence of each input variable on the predicted outcome of life satisfaction. To comprehensively evaluate the predictive performance of different models, this study constructs and compares four mainstream tree-based ensemble models: Random Forest, XGBoost, LightGBM, and CatBoost. All models are optimized through hyperparameter tuning using cross-validation combined with grid search to improve both predictive accuracy and generalization capacity. Finally, the models’ predictive performances are assessed on the testing set using several evaluation metrics, including Mean Absolute Error (MAE), Mean Squared Error (MSE), Root Mean Squared Error (RMSE), and the coefficient of determination (R²). The model demonstrating the best performance in predicting life satisfaction is identified. The overall research framework is presented in Fig 1.

The variable design in this study is based on the ‘2023 Report on Digital Information Divide Survey’ questionnaire developed by the NIA [34] and is further informed by prior research findings [18,31,32,39,66,67]. Relevant questionnaire items were integrated and categorized according to the research objectives. The variables encompass a wide range of dimensions related to the digital divide and quality of life, including digital device access and usage capabilities, technological self-efficacy, technology-related attitudes and perceptions, social capital, awareness and usage of AI services, digital literacy, and subjective life satisfaction. Each respondent was required to complete approximately 160 to 180 questionnaire items. According to the survey data, the ownership rate of personal computers (including desktop and laptop computers) among older adults was 49.8% (n = 432), with an average usage frequency of 2.66 days in the past month. In contrast, the ownership rate of smartphones reached 100%, with an average usage of 25.29 days per month. Consequently, this study focuses primarily on internet usage behaviors and related variables pertaining to smart devices (such as smartphones, computers, or tablets). To assess the internal consistency of the questionnaire, Cronbach’s alpha coefficient was employed. In general, an alpha value of 0.70 or above is considered indicative of acceptable internal consistency and reliability [68]. The questionnaire items, descriptive statistics, and results of the reliability analysis are presented in Table 2.

**Table 2:** Survey content on the digital information divide.

Measured Variable	Item Description	Mean	SD	Cronbach’s α
PC Usage Level	Days used on last month_PC (Desktop, Laptop)	2.656	6.772	–
Smart Phone Usage Level	Days used on last month_Smart phone	25.285	7.872	–
Tablet Usage Level	Days used on last month_Tablet	0.481	3.033	–
PC Use Ability	Ability to independently operate and manage a personal computer, including software installation and management, network connection, browser settings, peripheral use, file transfer, security protection, and document editing.	1.594	0.701	0.964
Mobile Device Use Ability	Ability to operate and manage smart devices, covering basic skills such as settings, network connections, file transfers, app management, security, and document processing.	2.180	0.777	0.927
Search, Email Content Service	Usage frequency of digital services through smart devices: online information/news search, email, media content browsing (movies, music, e-books), and educational digital content (e-learning, lectures).	1.575	0.472	0.776
Social Networking and Sharing Services	Frequency of using social networking and information-sharing services via smart devices: social networking services (SNS), instant messaging apps, personal blogs, online communities (forums, groups), and cloud services (e.g., Google Drive, OneDrive).	1.429	0.420	0.836
Lifestyle Services	Frequency of using digital lifestyle services: information services (weather, traffic, health, parenting), e-commerce, financial services (banking, investments, insurance), and government services (social security, taxation, medical appointments, e-government).	1.616	0.544	0.842
Information Production and Sharing	Frequency of content creation, editing, and information sharing using digital tools: producing or editing content (writing, video editing, graphic design) and sharing information (news, knowledge, videos, images) via social media, blogs, or links.	1.391	0.553	0.815
Online Networking	Frequency of using digital tools to maintain and build social relationships: maintaining contact with existing relations (family, friends, colleagues) and initiating new relationships through digital means.	1.622	0.568	0.715
Digital Social Participation Service	Frequency of participating in civic and public affairs via digital platforms: expressing opinions on social/political issues, submitting suggestions/feedback to government, online donations or volunteer activities, and engaging in digital civic actions (petitions, polls).	1.293	0.475	0.914
Online Economic Activity Service	Frequency of engaging in digital economic activities: job seeking, vocational training, entrepreneurship promotion, financial investments, gig economy activities, and cost-saving consumption strategies through online platforms.	1.324	0.490	0.905
Digital Supporter	Primary methods of resolving digital device issues: self-resolution, online tutorials, assistance from family or friends, social networks, or professional support services.	2.294	0.571	0.724
Attitude toward Digital Technology	Subjective attitudes and perceptions toward digital technologies, including emotional, cognitive, and behavioral dimensions: perceived usefulness, convenience, personal benefits, and willingness to use.	2.763	0.602	0.845
Digital Devices Self-efficacy	Self-efficacy and willingness to use digital devices: confidence in learning and using devices, ability to quickly adapt to new technologies, and willingness to adopt them.	2.129	0.711	0.884
Social Capital	Perceived access to social resources and support through personal networks: problem-solving support, advice-seeking, private discussions, collective action, and delegation of responsibilities; also includes perceived social connectedness and community engagement.	2.716	0.480	0.863
AI Service Awareness	Awareness of AI applications in various service contexts, including document writing, information retrieval, creativity, finance, social communication, healthcare, home automation, media, transportation, and education.	1.732	0.294	0.881
AI Assistance in Services	Degree of actual use of AI technologies for assistance in daily life or work across multiple service domains, including document writing, information retrieval, creativity, finance, social communication, healthcare, home automation, media, transportation, and education.	2.425	0.675	0.930
AI Perception	Cognitive and affective evaluations of AI technologies and their impacts on personal life, society, and human relations: positive perceptions (convenience, economic opportunities, improved services) and concerns (job displacement, privacy, misinformation).	2.833	0.422	0.793
Digital Information Literacy	Competence in acquiring, managing, processing, and safeguarding information in digital environments: digital operational skills, information judgment, privacy/security awareness, civic engagement, and digital content creation/application abilities.	2.052	0.884	0.968
Life Satisfaction	Overall subjective assessment of life quality, based on satisfaction with life circumstances, goal attainment, and life reflections.	2.521	0.491	0.788

### Dependent Variable

The dependent variable, life satisfaction, is measured based on Neto’s [35] conceptualization, which captures individuals’ cognitive evaluations of their overall life satisfaction. Specifically, this variable is assessed using a five-item scale on a four-point Likert scale (ranging from 1 = “strongly disagree” to 4 = “strongly agree”). The reliability analysis indicates that the scale demonstrates acceptable internal consistency, with Cronbach’s alpha coefficient of 0.788. The mean score for life satisfaction among the study sample is 2.521.

### Independent Variables

In multivariable modeling using machine learning, an excessive number of variables may lead to model overfitting, reduce interpretability, and hinder the practical applicability of model deployment. To address this issue, this study employs the feature importance analysis mechanism of the XGBoost model to select a representative subset of predictors from a broad set of digital divide-related variables that are most relevant to predicting life satisfaction among older adults. Initially, the study excluded variables related to personal identification and those with a high proportion of missing data from the original dataset of 2,300 observations, resulting in a refined set of 151 candidate independent variables for modeling. Subsequently, XGBoost’s built-in feature importance evaluation method was applied to compute three key importance metrics for each variable (Fig 3):

**Fig 2:**
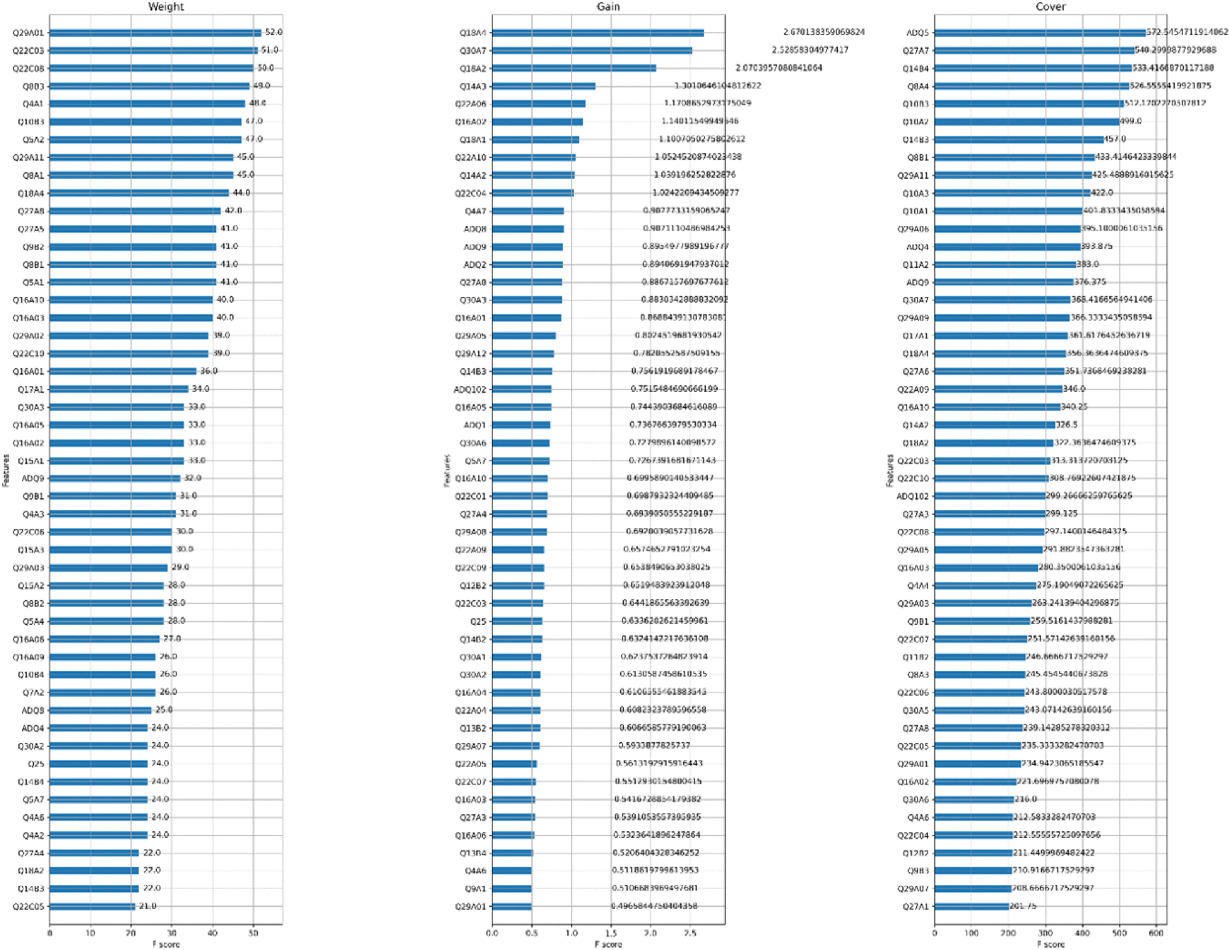
Importance of all independent variables.

Weight: The number of times a variable is used for tree splits.

Cover: The number of data instances separated by the variable during tree segmentation.

Gain: The average reduction in training loss achieved when a variable is used for splitting.

By calculating the feature importance indices (i.e., weight, cover, and gain) using the XGBoost model, this study identified the top 50 digital divide-related variables with high predictive importance for life satisfaction among older adults, as illustrated in Fig 2. Given that each importance metric captures different aspects of a variable’s contribution, relying on a single metric—as commonly seen in previous studies—may result in information loss or biased feature selection [69,70]. To address this limitation, this study applied Min-Max normalization to standardize the three importance metrics and subsequently computed a composite importance score through weighted averaging, thereby identifying variables that consistently demonstrated high importance across multiple dimensions. For example, the composite importance score for variable Q18A4 was calculated as follows: importance score = (0.846 + 1.0 + 0.622)/ 3 = 0.823. Based on the ranking of composite importance scores, the top 15 variables were selected to form the optimal feature subset for subsequent predictive modeling and interpretability analysis. Detailed results are presented in Table 3.

**Table 3:** Explanatory variables.

No.	Variable Name	Item Description
1	Q18A4	Digital Device Self-Efficacy_4) I want to use digital devices more frequently.
2	Q10B3	Usage of Lifestyle Services on Mobile Devices_3) Financial transaction services.
3	Q30A7	Digital Information Literacy_7) When my rights (e.g., reputation, copyright) are violated, I know how to report it on portals or social media.
4	Q29A11	Digital Information Literacy_11) I can use online simplified payment tools (e.g., Naver Pay, Kakao Pay) for shopping.
5	Q22C03	Degree of AI Assistance in Services_3) Services that use generative AI for content creation.
6	Q18A2	Digital Device Self-Efficacy_2) I am confident in using digital devices.
7	Q8B1	Usage of Search, Email, and Content Services on Mobile Devices_1) Information and news searches.
8	Q22C08	Degree of AI Assistance in Services_8) Media-related AI services.
9	Q16A10	Social Capital_10) Communication always allows me to meet new friends.
10	ADQ9	Monthly Household Income.
11	Q29A01	Digital Information Literacy_1) I can install, delete, upgrade programs, or copy, delete, move, and modify files and folders on a computer.
12	Q27A8	AI Perception_I believe AI helps improve interpersonal relationships.
13	Q14B3	Online Economic Activity Participation on Mobile Devices_3) I have used the internet to access information or financial services that help increase (or maintain) my income.
14	Q14B4	Online Economic Activity Participation on Mobile Devices_4) I have participated in cost-saving activities via the internet (e.g., group purchasing, overseas direct purchasing, price comparison, etc.).
15	Q17A1	Attitude Toward Digital Technology_1) I believe digital technology is useful.

### SHAP analysis

To further elucidate the key factors influencing life satisfaction among older adults and to explore their marginal effects, this study adopts the SHAP (SHapley Additive Explanations) method to systematically evaluate the contribution of each explanatory variable to the model’s output. SHAP is widely recognized for its local interpretability, as it reveals both the direction and magnitude of a variable’s contribution to model predictions across different values [64]. As shown in the SHAP summary plot (Fig 4), the distribution and gradient trends of the top 15 important variables are visualized. Overall, the variables that rank highly in the XGBoost model also exhibit substantial impact intensity and a generally positive effect on life satisfaction in SHAP visualization. A detailed interpretation based on variable dimensions is provided below.

First, variables related to digital technology self-efficacy demonstrate significant positive effects on life satisfaction. Specifically, higher values of Q18A4 (represented by red dots clustered on the right side of the plot) indicate that older adults who are more willing to engage with digital technologies tend to report higher levels of life satisfaction. Similarly, Q18A2 also shows a positive effect, albeit with a slightly weaker intensity, suggesting that subjective motivation to use digital technologies may exert a greater influence on well-being than actual skills alone. Although the digital technology attitude variable (Q17A1) shows relatively limited importance in the model, its positive distribution pattern implies that older adults with favorable attitudes toward digital technologies are more likely to experience a stronger sense of achievement and belonging in digital environments.

Second, the variable representing social participation and interaction capacity (Q16A10) exhibits a typical gradient pattern in its SHAP value distribution, with higher scores corresponding to pronounced positive effects. This finding suggests that active social interactions and relationship-building effectively enhance older adults’ emotional attachment to life and their perceived social support, making it a critical social determinant of life satisfaction.

Third, regarding basic digital information literacy skills, both Q10B3 (mobile financial service usage) and Q8B1 (mobile information search) display consistently positive effects on life satisfaction. These results highlight the importance of basic digital competencies in supporting older adults’ independent living capabilities. Although the SHAP values for Q14B3 and Q14B4 (engagement in online income generation and cost-saving activities) are relatively smaller, they still show positive trends, suggesting that participation in digital economic activities may enhance older adults’ sense of control over their lives. In contrast, higher-order digital operation skills, such as Q30A7, Q29A11, and Q29A01, exhibit more concentrated SHAP value distributions with limited impact intensity. This indicates that such advanced digital skills may function more as adaptive competencies rather than core drivers of life satisfaction.

Fourth, within the dimension of AI Assistance in Services and perception, variables such as Q22C03 (assistance from generative AI services) and Q22C08 (assistance from AI media services) predominantly display positive SHAP values, suggesting that the actual utility of AI services contributes positively to life satisfaction. By comparison, the effect of Q27A8 (perception that AI improves interpersonal relationships) is relatively weaker, implying that the quality of actual AI service experiences has a more direct and meaningful impact on well-being than mere cognitive perceptions of AI.

Finally, with respect to household economic status, the variable ADQ9 (monthly household income) shows a clear concentration of positive SHAP values among high-income samples, further confirming that household income remains a consistent and significant external determinant of subjective well-being, serving as an essential material foundation for life satisfaction.

To gain a deeper understanding of the linear relationships between each explanatory variable and the model output (life satisfaction), this study used SHAP dependency plots to reveal how changes in the values of each variable affect the prediction results. In the dependency plots, the SHAP value on the Y-axis represents the extent to which a variable either increases or decreases the predicted life satisfaction score at a given value of that variable (plotted on the X-axis, represented by either raw values or ordinal codes). The color gradient indicates the values of interacting features, capturing potential interaction effects. By examining the dependency plots, it is possible to analyze the magnitude and direction of the effects of different variable values on life satisfaction. As shown in Fig 5, the key analytical findings are summarized as follows:

**Fig 3:**
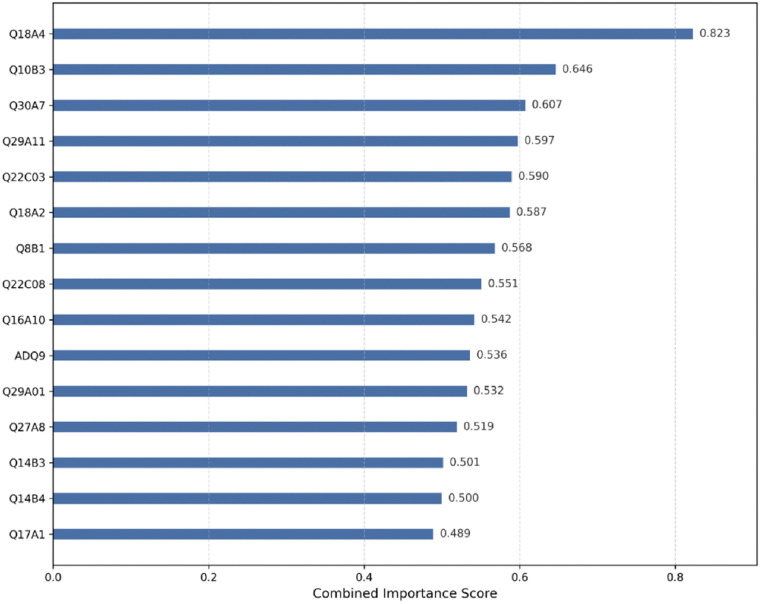
Importance of the first 15 explanatory variables.

**Fig 4:**
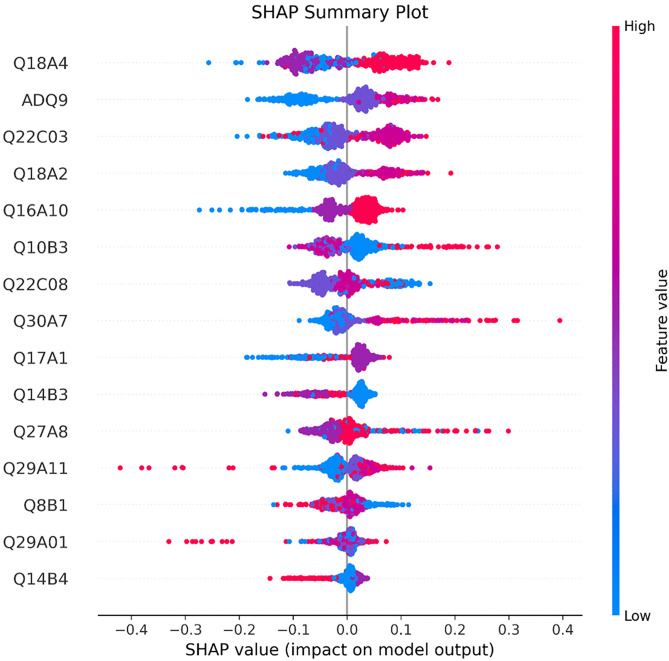
SHAP analysis: Summary plot.

**Fig 5:**
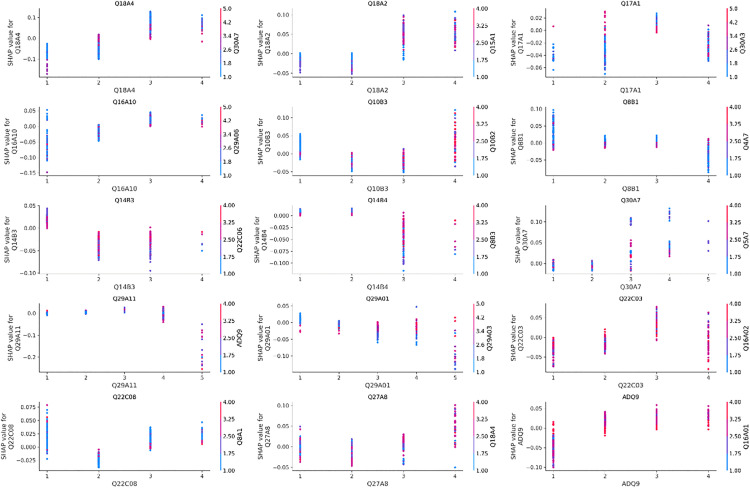
Dependency Plot.

First, variables related to digital technology self-efficacy and attitudes (Q18A4, Q18A2, and Q17A1) exhibit significantly positive SHAP values at higher scores, indicating that older adults’ confidence in and positive attitudes toward digital technologies can substantially enhance their life satisfaction.

Second, within the dimension of social capital (Q16A10), higher measurement scores are consistently associated with stronger positive SHAP values. This finding underscores the sustained positive impact of frequent social interactions and interpersonal engagement on life satisfaction, highlighting social capital as an essential source of social support.

Third, most variables related to digital information literacy (e.g., Q10B3, Q30A7, Q29A11, and Q8B1) display predominantly positive SHAP values at higher score levels (such as 4 or 5), suggesting that digital literacy plays a broad and direct role in enhancing well-being. In particular, professional digital competencies and participation in digital economic activities emerge as prominent influencing factors.

Fourth, variables related to AI-assisted service experience and perception (Q22C03 and Q22C08) generally exhibit positive SHAP values at high levels of agreement. This suggests that older adults who recognize the practical utility and social value of AI services tend to report higher levels of life satisfaction, reflecting the emerging role of digital service quality in shaping psychological well-being.

Fifth, as indicated by the economic status variable (ADQ9), older adults with better economic conditions exhibit higher SHAP contribution values, further confirming that financial resources serve as a critical external safeguard for life satisfaction among older adults.

### Model Evaluation

This study employed the following tree-based ensemble learning methods: Random Forest, as well as three gradient boosting models—XGBoost, LightGBM, and CatBoost. To ensure model stability and predictive performance, five-fold cross-validation was applied in conjunction with grid search (GridSearchCV) for hyperparameter optimization, through which the optimal model configurations were identified.

To comprehensively evaluate the performance of each tree-based ensemble model in predicting life satisfaction among older adults, this study adopts several commonly used regression evaluation metrics, including Mean Absolute Error (MAE), Mean Squared Error (MSE), Root Mean Squared Error (RMSE), and the Coefficient of Determination (R²) [20,71].

- **MAE**

MAE measures the average absolute difference between the predicted and actual values. It is calculated as follows:


$$\begin{array}{c} MAE=\;\frac{\text{1}}{n}\sum {\mathrm{\backslash nolimits}}_{t=\text{1}}^{n}\left|y_{i}-{\hat{y}}_{i}\right| \end{array}$$

Where $y_{i}$ denotes the actual value, ${\hat{y}}_{i}$ represents the predicted value, and n is the sample size. Lower MAE values indicate smaller prediction errors.

- **MSE**

MSE computes the average of the squared differences between predicted and actual values, placing greater emphasis on larger errors.MSE can be calculated as


$$\begin{array}{c} MSE=\;\frac{\text{1}}{n}\sum {\mathrm{\backslash nolimits}}_{i=\text{1}}^{n}{\left(y_{i}-{\hat{y}}_{i}\right)}^{\text{2}} \end{array}$$

- **RMSE**

RMSE is the square root of MSE, providing an error measure in the same units as the target variable. Like MSE, it is sensitive to larger errors. Lower RMSE values indicate better model performance.RMSE can be calculated as


$$\begin{array}{c} RMSE=\;\sqrt{\frac{\text{1}}{n}\sum {\mathrm{\backslash nolimits}}_{i=\text{1}}^{n}{\left(y_{i}-{\hat{y}}_{i}\right)}^{\text{2}}} \end{array}$$

- **R2**

R² assesses the proportion of variance in the dependent variable explained by the model. It ranges from 0 to 1, with higher values indicating better model fit:


$$R^{\text{2}}=\text{1}-\frac{\sum_{i=\text{1}}^{n}{\left(y_{i}-{\hat{y}}_{i}\right)}^{\text{2}}}{\sum_{i=\text{1}}^{n}{\left(y_{i}-\overset{―}{y}\right)}^{\text{2}}}$$

where $\overset{―}{y}$ denotes the mean of the actual observed values. A higher R² value reflects stronger explanatory power regarding the variance in life satisfaction.

Using these four metrics, the study evaluates and compares four tree-based ensemble models: Random Forest, XGBoost, LightGBM and CatBoost. The evaluation results are summarized in Table 4. Overall, the performance differences among the models in terms of MAE, RMSE, and R² are relatively small. However, CatBoost demonstrates superior performance in minimizing prediction errors and maximizing model fit, while also offering high stability and generalizability. Therefore, CatBoost is selected as the benchmark model for subsequent interpretability analyses.

**Table 4:** Model Performance and Hyperparameter Settings.

Model	BestParams	MSE	RMSE	RMSE 95% CI	MAE	R2
RandomForest	{‘max_depth’: 10, ‘min_samples_split’: 5, ‘n_estimators’: 100}	0.2149	0.4636	0.4399-0.4882	0.4305	0.1208
XGBoost	{‘learning_rate’: 0.01, ‘max_depth’: 3, ‘n_estimators’: 200}	0.2032	0.4508	0.4299-0.4715	0.4282	0.1686
LightGBM	{‘learning_rate’: 0.01, ‘max_depth’: −1, ‘n_estimators’: 200}	0.2046	0.4524	0.4273-0.4763	0.4214	0.1628
CatBoost	{‘depth’: 4, ‘iterations’: 100, ‘learning_rate’: 0.1}	0.2008	0.4481	0.4198-0.4765	0.4050	0.1785

**Fig 6:**
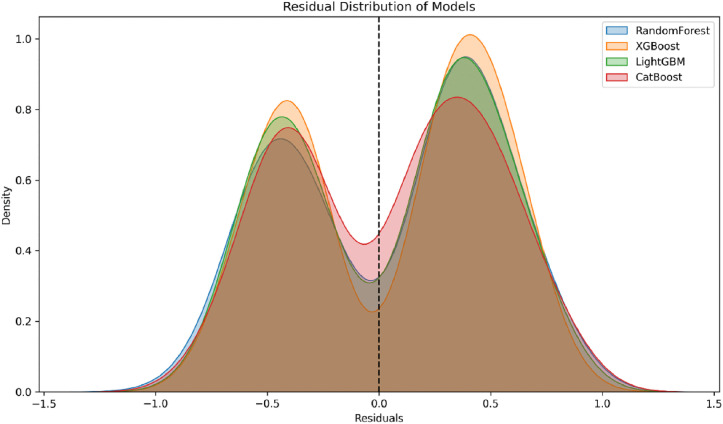
Residual Density Distributions of Four Models.

To further compare the predictive accuracy and error structures of the models, Fig 6 presents the residual density distributions of the four models on the test set. Overall, the residual density curves of all four models exhibit an approximately symmetric shape, without notable skewness or heavy-tailed patterns, consistent with the expected normality assumption. This indicates that the predictive errors do not display systematic bias and that the models achieve generally stable fits. In comparison, the CatBoost model demonstrates more balanced peak heights and stronger symmetry, suggesting a higher concentration of errors around zero. This implies that most prediction errors are relatively small, reflecting superior generalization performance. The residual distribution analysis therefore provides further support for the superior performance of CatBoost on the dataset used in this study.

**Fig 7:**
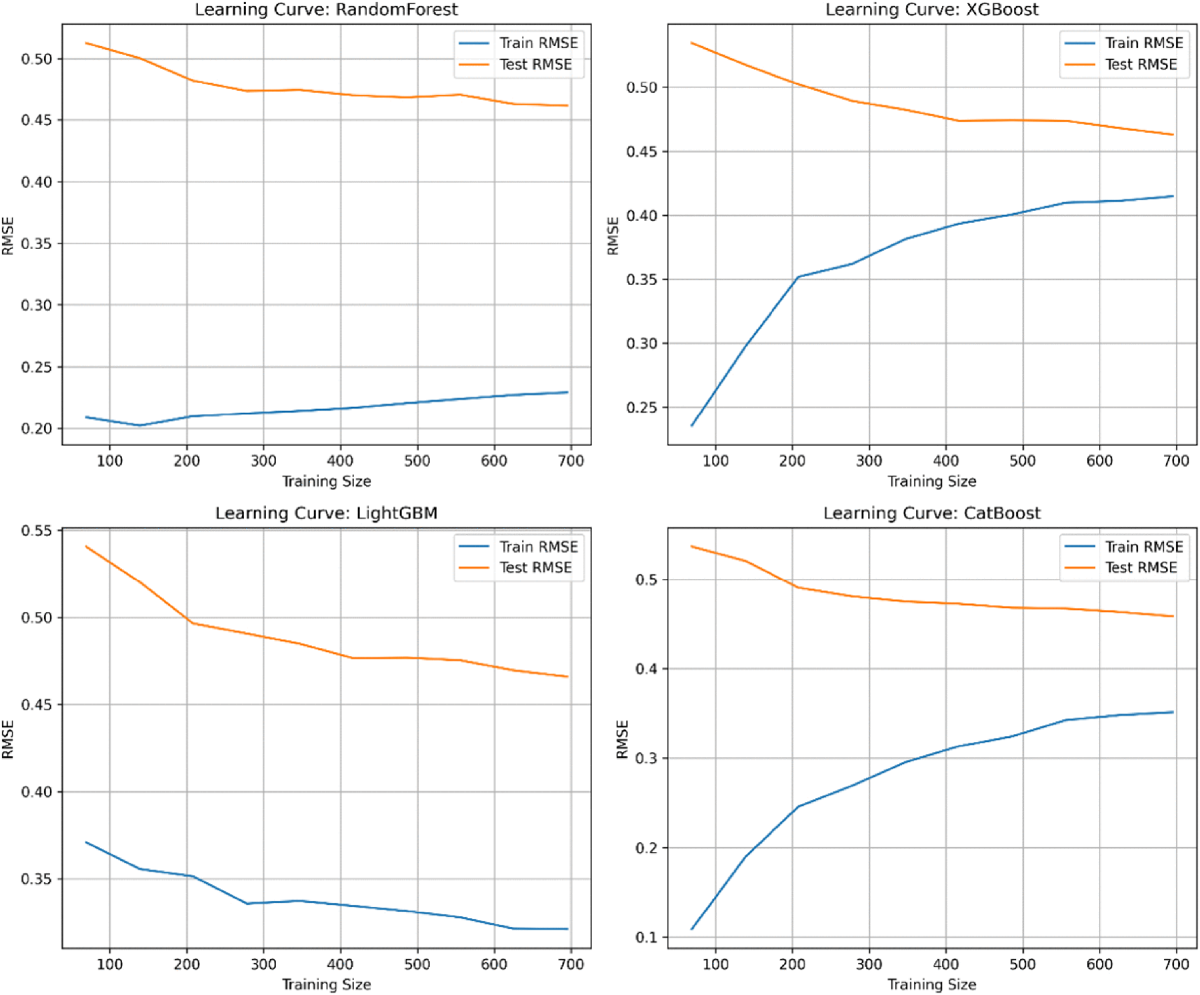
Learning curves of each model.

This study plotted learning curves to evaluate the bias–variance performance of the models (Fig 7). As the number of training samples increased, the test errors of all models exhibited a downward trend. Among them, XGBoost and CatBoost achieved relatively lower test errors and demonstrated smaller gaps between training and test errors, indicating strong generalization capacity and a favorable bias–variance balance, making them well-suited for practical prediction tasks.

Although early stopping is commonly used to mitigate overfitting in boosting models, it was not applied in this study. Instead, model complexity was effectively controlled through five-fold cross-validation, hyperparameter tuning (e.g., max_depth, learning_rate, and n_estimators), and learning-curve diagnostics. These procedures provided strong safeguards against overfitting while ensuring consistency across models, as all algorithms were trained with a fixed number of iterations to maintain comparability. Therefore, we believe that omitting early stopping does not compromise the robustness of our results, although future research may incorporate early stopping strategies to further improve computational efficiency.

### Results and discussion

This study aims to analyze the digital divide factors that influence life satisfaction among older adults. To identify the key predictors of life satisfaction, an XGBoost-based feature importance analysis was conducted on the input variables. The results indicate that the most influential factors primarily cluster around the following dimensions: digital technology self-efficacy and attitudes (Q18A2, Q18A4, Q17A1); social capital (Q16A10); digital information literacy (Q10B3, Q30A7, Q29A01, Q29A11, Q8B1, Q14B3, Q14B4); AI service usage and perception (Q22C03, Q22C08, Q27A8); and demographic economic status (ADQ9). These findings suggest that older adults’ digital self-efficacy, social relationships, digital literacy, and engagement with AI technologies serve as critical predictors of their life satisfaction.

To further enhance the interpretability of the model and ensure scientific rigor in variable selection, this study employs SHAP for model interpretability analysis. SHAP dependency plots were generated to uncover the specific nonlinear relationships between variable values and the predicted outcomes. The results demonstrate that variables related to digital technology self-efficacy and information technology capabilities exert particularly strong influences on life satisfaction, highlighting them as priority targets for digital inclusion policy interventions. A comprehensive summary and interpretation of the key findings are presented below.

First, digital technology self-efficacy and attitudes toward technology are significant predictors of life satisfaction among older adults. Confidence in digital technology reflects an individual’s sense of self-efficacy. Prior research has identified technological self-efficacy as a key competency for adapting to digital environments and effectively utilizing digital devices [18]. When older adults possess strong confidence in their technological abilities, they are more willing to adopt and actively use digital devices, facilitating smoother integration into the digitalized society. As older adults become more familiar with digital technologies, they are better positioned to access various digital healthcare services and other forms of social support provided by governments [72]. This not only improves their individual quality of life but also alleviates the caregiving burden on family members, thereby enhancing overall household well-being. In particular, according to the Theory of Innovation Resistance [73], high levels of self-efficacy are instrumental in the adoption of new skills [74]. When individuals are confident in their ability to learn and apply new skills, their resistance to adopting such skills decreases [75]. Therefore, in the era of digitalization, self-efficacy and attitudes toward digital technologies represent essential factors in predicting life satisfaction among older adults and should be given considerable attention in relevant research and policy design.

Second, social capital emerges as a crucial factor in explaining variations in life satisfaction among older adults. Social support, quality of interpersonal relationships, and frequency of social interactions are all important factors affecting life satisfaction in later life. Previous research has consistently shown that close connections with family members and broader social networks serve as key predictors of life satisfaction among older adults [76]. Older individuals who maintain stable and positive social relationships tend to report higher levels of subjective well-being [77]. In contrast, those experiencing limited social interactions or prolonged social isolation generally exhibit lower levels of life satisfaction [78,79]. In the context of increasing digitalization, several studies have also found a significant association between internet usage and life satisfaction among older adults [32,80]. The present study further confirms these findings, demonstrating that interpersonal relationships with family and broader social networks remain essential predictors of life satisfaction in old age.

Third, digital information literacy—particularly older adults’ ability to use and engage with digital technologies—emerges as a critical factor in explaining differences in life satisfaction. Competencies related to operating and understanding digital devices (such as smartphones and computers) and their functions (including app usage, information retrieval, and online communication) are closely associated with enhanced feelings of control over one’s life and stronger social connectedness, both of which contribute to higher levels of life satisfaction [81,82]. Furthermore, participation in daily activities through digital means—such as online shopping, financial services, telemedicine, and social media interaction—not only enables older adults to overcome physical and informational barriers but also facilitates their integration into an increasingly digital society [31]. Such digital competencies allow older adults to maintain their independence and social engagement in the digital era, which in turn fosters greater subjective well-being and life satisfaction. Notably, activities that involve increasing income or reducing expenses through digital platforms demonstrate the direct economic utility of digital tools in enhancing subjective well-being among older adults [81,83]. These findings indicate that digital information literacy should not merely be regarded as a measure of technological adaptation, but rather as a key variable influencing social participation, economic independence, and psychological well-being in later life.

Fourth, the findings further support prior research on the relationship between the use of emerging technologies and life satisfaction [33,84]. The results show that older adults’ use and perception of AI services are highly predictive of their life satisfaction. Specifically, those who perceive generative AI creative services and AI-based media services as beneficial tend to report substantially higher levels of life satisfaction. This suggests that when AI technologies are designed to be user-friendly and highly practical in daily life, they can effectively reduce older adults’ sense of alienation from digital environments, thereby enhancing their sense of control and subjective well-being [82]. Moreover, older adults who perceive AI as helpful in improving interpersonal relationships also report higher life satisfaction. This finding is particularly salient within the social context of the COVID-19 pandemic [79], during which social isolation and physical distancing measures significantly restricted in-person social interactions. In such circumstances, digital technologies—including video calls and AI-assisted communication tools—became essential channels for maintaining interpersonal connections and accessing social support [85]. Therefore, the results of this study not only highlight the positive impact of older adults’ subjective experiences with AI services on life satisfaction but also provide further evidence of the critical role of ‘technology accessibility’ and ‘perceived technological benefits’ in promoting active aging.

Finally, consistent with the findings of Joshanloo and Jovanović [86], Blanchflower [87] and Saldivia et al. [88] this study confirms that household economic characteristics, a key demographic factor, also influence life satisfaction. Older adults with limited financial resources often lack the necessary means and opportunities to learn and adapt to digital technologies, resulting in greater constraints on their social interactions, access to information, and ability to engage in everyday activities. Socioeconomically disadvantaged groups are more likely to face substantial challenges in adapting to digital transformation processes [89]. This argument is further supported by the results of the present study, indicating that economic conditions must be carefully considered when predicting older adults’ ability to adapt to digitalization. In particular, policies promoting digital inclusion should take into account economic disparities to effectively address the digital divide and enhance life satisfaction among older adults.

It is also worth emphasizing that although this study focuses on older adults in the South Korean context, its findings carry important comparative and heuristic implications on a global scale. In recent years, international scholarship on the relationship between the digital divide and the well-being of older adults has grown considerably. For example, Charness and Boot [38] found that in North America, older adults’ confidence in using digital technologies was significantly associated with their level of social integration, a result that resonates with this study’s identification of “digital technology self-efficacy” as a core predictor of life satisfaction. Similarly, Yang et al. [80] demonstrated that in China, older adults improved their information management capacities through online services, thereby enhancing subjective well-being—an outcome consistent with the positive effects of digital information literacy (e.g., online financial behavior and information discernment) revealed in this study. In Latin American countries, Sen et al. [90] reported that accessibility and ease of use of digital tools were especially critical for improving the quality of life of socioeconomically disadvantaged older adults, corroborating this study’s identification of “economic status” as a major constraint on digital adaptation.

Cross-national comparative studies further indicate that differences in social institutions, cultural values, and family structures shape the actual significance and modes of digital technology use among older adults [91]. For instance, In European countries, government-led digital training initiatives and nationwide digital inclusion programs have contributed to narrowing intergenerational gaps [92,93]. By contrast, in East Asian contexts, family members play a more prominent role in guiding older adults’ digital adaptation [94]. Compared with these regions, South Korea has achieved a high level of digital government development, digital disparities among older adults remain substantial [34]. This makes “perceived technological benefits” and “self-efficacy” particularly relevant entry points for practical interventions.

Therefore, this study not only addresses the scholarly concern regarding the relationship between the digital divide and life satisfaction among older adults but also provides representative empirical evidence from South Korea, one of the world’s fastest-aging societies. Future research could further extend cross-cultural validation in multi-country settings to examine how different social structures, welfare regimes, and stages of digital development shape pathways of digital empowerment for older adults.

## Conclusion

This study investigates the mechanisms underlying life satisfaction among older adults, with a particular focus on the role of digital divide-related factors. By integrating feature importance analysis using the XGBoost model with SHAP (SHapley Additive Explanations) analysis, the study develops a predictive and interpretable analytical framework. The results systematically identified digital technology self-efficacy and attitudes, social capital, digital information literacy, use and perception of artificial intelligence services, and household economic characteristics as important predictors of life satisfaction among older adults. In order to build a life satisfaction prediction model, we evaluated four tree-based ensemble learning algorithms: Random Forest, XGBoost, LightGBM, and CatBoost. In terms of prediction accuracy, the CatBoost model performed well, indicating that this decision tree-based ensemble method is the best model for predicting life satisfaction among older adults. The CatBoost model demonstrated superior performance in the cross-sectional analysis and outperformed the other algorithms considered in this study, confirming its effectiveness in modeling complex, nonlinear relationships in social science research on aging and digitalization.

The academic contributions of this study are reflected in the following aspects. First, in contrast to prior research that has predominantly relied on linear regression models, variable control methods, or mediation analysis, this study introduces a machine learning-based feature importance ranking mechanism alongside the SHAP interpretability framework. This approach enhances the ability to capture complex, nonlinear relationships among high-dimensional variables while also improving model transparency and the interpretability of the findings. At the same time, it complements existing theoretical frameworks on digital technology acceptance and use—such as the Technology Acceptance Model (TAM), the Unified Theory of Technology Acceptance and Use (UTAUT), and Jan van Dijk’s multidimensional framework of the digital divide. By empirically identifying and validating the key variables and mechanisms emphasized in these theories, our study strengthens the integration of theory-driven and data-driven approaches. Second, compared to traditional machine learning algorithms, ensemble learning methods demonstrate superior effectiveness in predicting digital divide-related factors affecting life satisfaction among older adults. In particular, the CatBoost model exhibits outstanding performance in the cross-sectional analysis, outperforming the other algorithms considered in this study. Furthermore, feature importance analysis reveals that variables related to digital technology self-efficacy and attitudes, social capital, digital information literacy, and AI service usage and perception are among the most salient predictors of life satisfaction among older adults. These findings not only corroborate theoretical claims about the importance of digital literacy and social participation but also provide fine-grained empirical evidence through methodological innovation. In doing so, this study offers both theoretical insights and methodological contributions to the fields of active aging and digital empowerment.

At the practical level, the findings of this study offer valuable policy implications. First, the analysis based on XGBoost and SHAP demonstrates that digital self-efficacy, social capital, and digital information literacy are key predictive variables for life satisfaction among older adults—particularly through the dominant nonlinear effects observed in SHAP value decomposition. This evidence offers a robust empirical foundation for governments and social organizations to design targeted and personalized digital empowerment policies. For example, prioritizing efforts to enhance older adults’ sense of control over digital devices, their autonomous usage capabilities, and their acceptance of AI services is essential for narrowing the digital divide, fostering digital engagement, and ultimately improving subjective well-being among older populations. Second, this study emphasizes that digital empowerment should not rely solely on government-led interventions but requires multi-stakeholder collaboration. Family members, as the immediate support system for older adults’ digital lives, can offer usage guidance, technical companionship, and emotional support in everyday interactions, forming a ‘digital intergenerational co-learning’ mechanism at the household level. Such as initiatives on ‘family co-learning programs’ or ‘intergenerational digital tutoring’, which reinforce intrafamilial knowledge transfer and emotional bonds. Family members can provide critical contextual assistance and psychological support during the early stages of digital technology adoption, helping to mitigate technology anxiety and usage barriers. At the same time, service providers—especially financial institutions, telecommunications operators, and health management platforms—should assume greater responsibility for offering age-friendly digital services. For instance, financial institutions may lower the technological barriers for older users by simplifying interface design, establishing offline digital consultation counters, or developing voice-guided applications, thereby enhancing users’ sense of security and trust. Moreover, key variables identified through SHAP analysis, such as prior AI service experience and technology-related perceptions, provide valuable user insights for enterprises in designing ‘silver-friendly’ intelligent products. Finally, the explanatory predictive framework proposed in this study has strong external applicability and can be extended to other disadvantaged groups (e.g., people with disabilities, low-income rural populations, and individuals with limited educational backgrounds) in research on digital access and well-being enhancement. This framework thus offers both theoretical support and practical decision-making tools for advancing universal digital inclusion and equity.

This study still has the following limitations. First, the study sample was limited to older adults with internet usage experience, thus excluding those who have never accessed or used digital devices. This sampling constraint may lead to an underestimation of the effects of the digital divide, particularly in terms of structural disparities in information accessibility and life satisfaction. Future research should broaden the scope of sample design by including non-internet users to more comprehensively capture the stratified characteristics of life satisfaction across different segments of the older population in digital environments. Second, while this study employed tree-based ensemble learning methods for predictive analysis, the sample size remained relatively limited. Although ensemble learning methods exhibit a certain degree of robustness under small-sample conditions, their optimal performance generally relies on larger datasets. Therefore, future research could leverage longitudinal or rolling survey datasets, such as the Digital Divide Survey, to conduct cross-period or pooled-sample analyses, thereby enhancing model stability and improving the robustness of the results. Third, the predictive analysis primarily relied on tree-based ensemble algorithms. Although these approaches effectively capture nonlinear feature importance, the absence of comparisons with other mainstream machine learning methods (e.g., support vector machines, neural networks) may constrain the comprehensiveness of model evaluation. Moreover, in terms of model interpretability, SHAP was the primary technique employed. While SHAP performs well in explaining the marginal contributions of variables, other explainable artificial intelligence (XAI) methods (such as LIME or counterfactual explanations) were not considered. Accordingly, future studies are encouraged to adopt a multi-algorithm validation framework and incorporate a broader range of XAI techniques to further enhance model transparency and interpretability. In addition, early stopping strategies were not employed in this study, as model complexity and overfitting were sufficiently controlled through cross-validation, hyperparameter tuning, and learning-curve diagnostics. While this ensured consistency across models, future work may incorporate early stopping to further enhance training efficiency. Finally, the geographic scope of the data must also be considered. This study drew upon samples from a single national context (South Korea), meaning that the findings are shaped by the country’s specific cultural, welfare, and digital development conditions. To enhance external validity, future studies should undertake comparative analyses across countries and regions, particularly those with markedly different institutional systems, levels of economic development, and cultural backgrounds. Such efforts would allow for testing the generalizability and robustness of the proposed model and conclusions in cross-cultural contexts.
